# The Remedy of Recognition an Imperative Duty

**Published:** 1917-01-20

**Authors:** 


### HOMELAND WORKERS IN WAR TIME.

### THE REMEDY OF RECOGNITION AN IMPERATIVE DUTY.

THE war has revolutionised many things, and there are few fields of work where the resulting changes have had more severe effects upon individual workers in responsible positions, than they have had on those working in the hospitals, and in some organisations for district and other nursing. Sometimes the strain has made itself grievously felt in the case of members of the medical staff of all ranks; of the matrons and lady superintendents; and of the secretaries and superintendents, where the individual has been endowed with special ability, great administrative gifts, and a conscientious desire to spend and be spent in securing the adequate treatment and comfort of the warrior and other patients. Just as the Victoria Cross may never come within the reach of the bravest man in the Navy or Army, however valiant a warrior he may be, so in our civil hospitals and arrangements for the treatment of the sick, there are only relatively few individuals, comparatively, who have encountered the special and wearing strain and continuous pressure of the insistent character we have in mind. The enormous increase in the number of hospitals and agencies of various kinds for the treatment of the wounded and the sick, and the special need of nursing provision outside hospitals for district and other cases, which, in the circumstances of the moment, do not find admission to a hospital, have created this ever-present strain.

We are led to call attention to this aspect of war work as it has affected, and still affects, some civil workers amongst the sick, because the services of the heroes and heroines in question are of the highest. It is remarkable that so far there has been an absence for them of honours and official recognition in the *Gazette*, though their services, on the merits, are comparable to and may even be in excess of those rendered by some types of workers at the Front, whose class has been continually honoured. In foreign countries the authorities are much better informed, or much quicker to realise onerous services, such as those referred to. We can only hope that before the war is over there may be instituted an Order of Special Devotion or Special Service to meet the claims of these splendid specimens of the national character in action, at its best, seeing that the extent of this service of self-sacrifice in civil life is probably infinitely greater, than ever before in the history of the nation.

There are a number of instances where members of the medical profession, surgeons for example, have continuously remained on duty—where operators were few and the cases great in number and vitally pressing—for practically the whole day and night, with only short intervals for necessary refreshment. These examples are most numerous, probably, at the Front, but the pressure in the Homeland, where at times the number of urgent cases admitted from the Front has been great and continuous, and the consequent strain on the surgeons and their immediate assistants has been onerous indeed. Amongst the T.F.N.S. Principal Matrons there are instances where, in addition to very onerous responsibilities attaching to the holder of this important office and to her continuous duties as matron of an important general hospital, in the most active voluntary hospital centres for war work, the Principal Matron has had to undertake and discharge heavy duties in connection with the new military hospitals and asylums containing 2,000 beds or more. Here the accommodation for patients may be divided into sections situated on various sites some distance apart from each other, and sometimes even a considerable train journey distance from the central hospital. We know of one, and there are probably others, where these sections amount to at least ten. In the course of our inspections we have been filled with admiration by the devoted service the Principal Matrons in question have rendered and are rendering at great physical expense to themselves, and to the everlasting honour of the nursing profession of which they constitute splendid types. The marvel is that some have been enabled, continuously without a breakdown, to discharge the numerous scattered, exacting, and pressing duties for which they are responsible. The same remarks apply in a few cases to the output called for on the part of the responsible head of a great organisation of district nurses, where the increased difficulties of work entailed by the causes we have indicated may have entailed a pressure of equal weight and strain.

Take, again, the case of the director or secretary-manager of the larger Metropolitan hospitals and of a few of the greater provincial general hospitals with medical schools. There are instances of magnificent service on the part of these laymen, which has enabled splendid provision to be made and an adequate staff of workers of all grades to be added, whereby many hundreds of additional beds have been placed at the disposal of the naval and military authorities, with the maximum of efficiency and despatch. Here, again, in the few cases we have in mind the strain upon the individual worker has been continuous and sometimes exacting. The outside world and, apparently, the Government authorities and their superiors, have so far failed to apprehend or have brought to their knowledge the generous and great services thus rendered.

These facts constitute the reasons which have moved us to indicate some notable instances of the personal service rendered by our Homeland workers in war-time. Some of them, we have reason to fear, may not be able, despite their resolute strength and courageous persistence in the good work, to continue it for an indefinite period. They will, we know, so long as the need exists, put into the effort all that is best and strongest in their nature. They so hope to continue, until the end of the war, the really splendid service they are rendering to the nation and the State. Is it impossible for those immediately responsible to recognise these zealous medical and civil administrators in the Homeland Service, by giving them equal recognition and encouragement, to that already extended in the official *Gazette*, to many other workers in all branches of the service? We believe not, because the omission has no apparent justification, except that which may rest upon an absence of fulness of knowledge in high places of the actual facts. Experience proves that, encouragement and recognition are the most efficacious stimulant and remedy possible for the zealous worker, who has no thought for self.

